# Bioactive Peptides from Fermented Foods: Production Approaches, Sources, and Potential Health Benefits

**DOI:** 10.3390/foods13213369

**Published:** 2024-10-23

**Authors:** Laryssa Peres Fabbri, Andrea Cavallero, Francesca Vidotto, Morena Gabriele

**Affiliations:** Institute of Agricultural Biology and Biotechnology, National Research Council, 56124 Pisa, Italyandrea.cavallero@ibba.cnr.it (A.C.); francesca.vidotto@ibba.cnr.it (F.V.)

**Keywords:** fermented foods, bioactive peptides, microbial fermentation, health benefits, functional foods, nutraceuticals, disease prevention

## Abstract

Microbial fermentation is a well-known strategy for enhancing the nutraceutical attributes of foods. Among the fermentation outcomes, bioactive peptides (BAPs), short chains of amino acids resulting from proteolytic activity, are emerging as promising components thanks to their bioactivities. Indeed, BAPs offer numerous health benefits, including antimicrobial, antioxidant, antihypertensive, and anti-inflammatory properties. This review focuses on the production of bioactive peptides during the fermentation process, emphasizing how different microbial strains and fermentation conditions influence the quantity and quality of these peptides. Furthermore, it examines the health benefits of BAPs from fermented foods, highlighting their potential in disease prevention and overall health promotion. Additionally, this review addresses the challenges and future directions in this field. This comprehensive overview underscores the promise of fermented foods as sustainable and potent sources of bioactive peptides, with significant implications for developing functional foods and nutraceuticals.

## 1. Introduction

As consumers are becoming increasingly knowledgeable about health and nutrition, the interest in fermented foods providing a less processed, naturally based, and health-promoting option is growing accordingly [1,2]. The fermentation process consists of a microbial-driven enzymatic transformation of substrates derived from various foods, including dairy products, grains, vegetables, meat, and many others. The microbial activity results in the generation of several products such as amino acids, organic acids, short-chain fatty acids (SCFAs), and bioactive peptides (BAPs), which endow fermented foods with a wide range of bioactivities [3].

BAPs are peptides consisting of 2 to 20 amino acids originating from precursor proteins following an activation process involving multiple reactions such as chemical hydrolysis, enzymatic hydrolysis operated by proteolytic enzymes, or microbial fermentation by proteolytic bacteria [4,5]. The health-promoting potential of BAPs, of either plant or animal origin, has been recognized and linked with several useful bioactivities including antihypertensive, antioxidant, antimicrobial, and anti-inflammatory activities [4,6,7,8]. The most investigated BAPs are those present in milk and dairy products, legumes, cereals, meat, and fish [8].

Owing to their promising properties, research has focused on overcoming the challenges linked with the production and delivery of BAPs. Indeed, fermentation often results in low yields of BAPs, which can be increased by modulating physicochemical parameters such as time, temperature, and pH and using emerging green technologies [9]. Moreover, the stability and bioavailability of BAPs in biological systems can be increased through encapsulation strategies and physicochemical processes [10].

**Figure 1:**
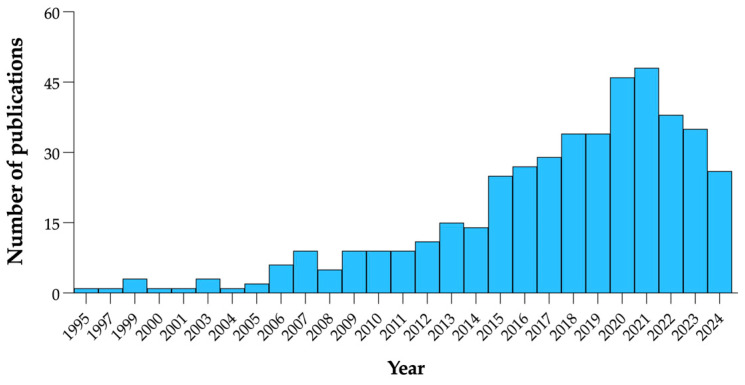
Bibliometric analysis of published scientific articles on ‘bioactive peptides from fermented foods’. The search was carried out in PubMed^®^ on articles from 1995 to 2024.

Noteworthily, the attention toward fermentation-produced BAPs has experienced a surge in recent years. As shown in Figure 1, the input ‘bioactive peptides from fermented foods’ generated 375 hits in PubMed, spread between the years 1995 and 2024, and reached a peak of publications in 2021. Thus, an up-to-date review of this relatively new field is offered.

The present review summarizes the available knowledge on BAPs originated in fermented foods, focusing on their generation, sources, and physiological effects and challenges and strategies to enhance their content and delivery. A better understanding of these aspects could open a path toward innovative food products that may contribute to improving human health and nutrition.

## 2. Fermentation Process for the Synthesis of BAPs

Fermentation is an ancient practice used across several cultures worldwide, from Asia to Europe and the Middle East, to preserve and enhance the nutritional value of foods [11]. This process not only alters the taste, smell, and texture of food but also improves its digestibility and nutritional profile [12]. By breaking down complex carbohydrates and proteins, fermentation increases the intestinal absorption of nutrients, vitamins, and minerals, making them more bioavailable [8,12,13]. Additionally, fermentation lowers the glycemic index of foods thanks to the activity of microbial enzymes such as maltase and α-amylase, which convert starches into simple sugars that are readily absorbed by microorganisms, resulting in a low carbohydrate content in the final product [14].

One of the most exciting outcomes of fermentation is the production of BAPs. These peptides are formed through proteolytic enzymatic reactions, where proteins are hydrolyzed into smaller aminoacidic chains with beneficial health effects [15]. Lactic acid bacteria (LAB) and some fungi such as *Aspergillus oryzae* are central to this biosynthetic process. The most cited LABs for their effectiveness in producing BAPs are *Lactobacillus helveticus*, *Lactobacillus delbrueckii* ssp. *bulgaricus*, *Lactococcus lactis* ssp. *diacetylactis*, *L. lactis* ssp. *cremoris*, and *Streptococcus salivarius* ssp. *Thermophilus* [16]. In addition to LAB, other microorganisms such as *Bacillus* species and certain fungi play a significant role in BAP production. For example, *Bacillus subtilis* and *Bacillus licheniformis* are known for their robust proteolytic activity, allowing them to hydrolyze proteins into bioactive peptides [17,18]. Fungi such as *Aspergillus oryzae*, *Candida lipolytica*, *Saccharomyces cerevisiae*, and *Rhizopus oligosporus*, among others, are also extensively used in BAP production due to their variety of enzymes, including protease, capable of breaking down specific types of proteins derived from plants or animals. Fungal strains exhibit a remarkable metabolic diversity, enabling them to utilize diverse carbon and nitrogen sources for the new synthesis of proteins and generation of BAP [19].

LAB species have a highly developed proteolytic system composed of cell envelope proteinases (CEPs), peptide transporters, and intracellular peptidases. CEPs initiate hydrolysis by cleaving proteins into peptides, which are then transported into the cell and further degraded into amino acids by various peptidases [20].

Four types of CEP genes (PrtB, PrtP, PrtR, and PrtH) have been characterized in *Lactobacillus* species [20,21]. As an example, *L. helveticus* is highly proteolytic due to the presence of up to four PrtH paralogs with different specificities, making it particularly efficient at generating diverse BAPs [21,22,23]. Several factors, including variations in CEP gene expression, mutations, and specific hydrolysis conditions, influence the variability in BAP profiles among LAB strains [24].

Besides the microbial species, to obtain peptides with enhanced bioactivity, it is crucial to carefully manage the choice of substrate and maintain favorable environmental conditions, including pH and temperature, throughout the fermentation process [8,25]. Additionally, the diversity of animal- and plant-derived proteins plays a significant role in determining the variety of BAPs produced and their bioactivities. For instance, BAP-rich fermented products such as camel milk, Panxian and Spanish dry-cured ham, natto, and tempeh have shown notable health benefits, including antioxidant, antihypertensive, antibacterial, and anti-inflammatory activities [26,27,28,29,30,31].

Microbial fermentation, in general, has emerged as an efficient and cost-effective way of producing BAPs. This process is more convenient compared to conventional enzymatic hydrolysis, being cheaper and simplifying production by avoiding the multiple purification steps through fermentation [32]. However, challenges like low peptide yields and lack of specificity remain obstacles in industrial applications. Ongoing research aims to optimize fermentation conditions, microorganism selection, and substrate management to enhance peptide bioactivity and maximize health benefits [8].

## 3. Fermented Foods as Sources of BAPs

### 3.1. Milk and Dairy Products

BAPs in milk play a crucial role in human health [33]. Milk proteins, primarily caseins (α-casein, β-casein, and κ-casein) and whey proteins (β-lactoglobulin, α-lactalbumin, and lactoferrin), are rich in BAPs released during fermentation and digestion [34]. The use of LAB for proteolytic activation enriches milk-derived foods with bioactive compounds [35,36]. *Lactobacillus*, *Lactococcus*, and *Streptococcus* species, and some yeast strains, such as *Saccharomyces cerevisiae/paradoxus* and *Kluyveromyces marxianus*, have been identified in fermented dairy products and demonstrate the ability to release BAPs with antioxidant, antihypertensive, and antimicrobial properties [37,38,39].

Ripening and in vitro digestion processes in dairy products like Parmigiano Reggiano (PR) cheese enhance the release of peptides with various health benefits, including antihypertensive, antioxidant, immunomodulatory, anti-inflammatory effects [40]. Similarly, kefir, prepared by adding 4% kefir grains to pasteurized whole milk and fermenting at room temperature for 24 h, led to a 19% inhibition of angiotensin-converting enzyme (ACE) activity and a 37 mmHg reduction in systolic arterial pressure in hypertensive rats [41]. Several studies have shown that milk fermented with *L. helveticus* is rich in two ACE inhibitory tripeptides, which exert an antihypertensive effect by reducing angiotensin formation [42,43,44]. Peptides derived from lactoferrin, a protein found in the milk of all mammals, have been shown to possess antimicrobial and immunosuppressive properties [45]. Antioxidant and ACE inhibitory peptides have also been identified in donkey milk by multidimensional liquid chromatography and nano high-performance liquid chromatography (HPLC)–high-resolution mass spectrometry [46].

The characteristics of peptides derived from milk can change depending on various factors, such as the hydrolysis method used and the animal producing the milk. For instance, bioactive proteins and peptides with different bioactivities can be achieved from buffalo, camel, goat, sheep, mare, and yak milk [47].

### 3.2. Meat

Meat and its derivatives are regarded as rich sources of BAPs due to their high protein content. Numerous BAPs have been found in meat and fermented meat products. Mora et al. [48] reported that the fermentation of a dry Spanish sausage, made with 75% lean pork and 25% pork back fat and supplemented with various additives (NaCl, lactose, dextrin, sodium caseinate, glucose, sodium ascorbate, sodium nitrite, and potassium nitrate), inoculated with *Lactobacillus pentosus* and *Staphylococcus carnosus*, and ripened in two stages (22 h at 15–20 °C followed by 43 days at 9 °C), generated peptides with ACE-inhibitory activity, such as YQEPLV, YQEPVLGPVR, and YQEPVLGPVRGPFPI, as well as the peptide YQEPVVLGPVRGPFPIIV, which is known for its antimicrobial activity.

Additionally, it was shown that camel meat sausages treated with a range of bacterial strains and cured for up to 28 days exerted a higher peptide concentration and an enhanced antioxidant and antihypertensive capacity compared to non-inoculated sausages, with results being positively affected by the ripening time [49]. The choice of the bacterial species and strain is vital for guaranteeing the best recovery of BAPs from meat, as demonstrated by Takeda et al. [50]. When studying the fermentation of sausages, they found that specific strains of *Lactobacillus sakei* and *Lactobacillus curvatus* exerted better proteolysis as well as antioxidant and ACE-inhibitory activities than both the non-treated and the type-strain-treated sausages. Furthermore, the generation of BAPs is also influenced by the kind of meat. For instance, uncured fallow deer sausages had a higher nutritional value than beef sausages, thanks to their greater peptide content, L-carnitine concentration, and antioxidant property [51].

### 3.3. Plant-Based Foods

Cereals are widely utilized to create novel foods and ingredients due to their high protein content (10–15%), making them a suitable substrate for probiotic fermentation [52]. Cereal proteins can be broken down by microbial enzymes during fermentation, producing BAPs [53]. Recent studies have highlighted the potential of fermented cereal products in improving human health.

Peptides from fermented rice displayed promising results in preventing cognitive decline and promoting brain health in mice by upregulating the levels of brain-derived neurotrophic factor [54]. Fermented oats, when processed with specific microbes like *L. plantarum* and *Rhizopus oryzae*, yielded more BAPs and showed higher ACE-I inhibitory activity than unfermented oats, which could be beneficial for hypertension [55]. Additionally, the less studied pseudocereals also benefit from fermentation. For instance, a 72 h solid-state fermentation of quinoa with *L. plantarum* K779 demonstrated an enhancement in its antioxidant and antihypertensive properties [56]. Likewise, fermenting amaranth using 40 strains of *Lactobacillus* species as starters at 30 °C for 16 h could potentially lead to the production of the cancer-preventive peptide lunasin [57].

Moreover, fermentation improves the nutritional qualities of legumes such as soybeans and red beans. For example, a solid-state fermentation of soybeans with *Bacillus amyloliquefaciens* led to a reduction in antinutritional factors and an increase in antioxidant activity [58]. Similarly, solid-state fermentation of red beans with *Cordyceps militaris* enhanced both protein digestibility and bioactivity [59]. These findings highlight the health benefits of fermented legumes, including their potential in managing conditions like obesity and diabetes.

Overall, microbial fermentation of cereals, pseudocereals, and legumes can significantly enhance their nutritional and functional properties, offering promising health promotion and disease prevention applications.

### 3.4. Marine Organisms

Fish and shellfish are renowned for their high protein content and are traditionally preserved through salting and fermentation. Fermented fish and shellfish, often used as savory condiments, are processed with starter cultures such as *L. plantarum*, *Lactobacillus brevis*, and *Bacillus mojavensis* [60,61,62].

Recent research has highlighted the bioactive potential of peptides derived from fermented fish and shellfish. For instance, fermentation of *Ruditapes philippinarum* clams with *Bacillus natto* for 36 h at 37 °C and pH 7.2 resulted in a peptide with notable ACE-I-inhibitory activity, which remained stable during gastrointestinal digestion. The inhibitory peptide was purified sequentially using ultrafiltration, gel filtration chromatography, and reverse-phase HPLC (RP-HPLC). This peptide not only enhanced nitric oxide (NO) release but also inhibited endothelin-1 secretion and scavenged reactive oxygen species, demonstrating its potential as a pharmaceutical agent [18]. Budu, a Malaysian fermented fish sauce made from anchovy (*Ilisha Melastoma*) through over 120 days of fermentation, produced BAPs with strong antioxidant properties, such as LDDPVFIH and VAAGRTDAGVH, identified through LC-ESI-TOF analysis. These peptides, rich in hydrophobic and acidic amino acids, are believed to contribute to the high antioxidant activity observed in Budu [63]. Similarly, Pekasam, a traditional Malaysian fermented fish product, was fermented with *L. plantarum* for 15 days at 27 °C, with a pH range of 4.9 to 5.6. The fermentation process yielded two novel antioxidant peptides, identified by HPLC connected to tandem mass spectrometry (LC/MS/MS), which exhibited significant radical scavenging activity, attributed to their high content of hydrophobic amino acids [64]. Furthermore, commercial Thai fermented shrimp pastes, aged for six months, were found to contain peptides with antioxidant and ACE-I-inhibitory activity, isolated through sequential anion exchange chromatography and solid-phase extraction using a C18 matrix [65].

In another study, Zebra blenny, fermented with *Bacillus mojavensis* A21 at 37 °C and 200 rpm for 4 to 48 h and then fractionated using Sephadex G-25 gel filtration and RP-HPLC, produced antimicrobial peptides identified by nano ESI-LC–MS/MS. These peptides demonstrated significant antibacterial activity against various pathogens, including *Micrococcus luteus* and *Escherichia coli* [62].

Overall, these studies illustrate the significant health benefits of BAPs from fermented fish and shellfish, including antimicrobial, antioxidant, and antihypertensive properties, suggesting their potential use in both food supplements and functional foods.

## 4. Bioactivities of BAPs Derived from Fermented Foods

BAPs originated in fermented foods perform several biological functions as highlighted by extensive research. In the next sections, the antimicrobial, antihypertensive, antioxidant, and anti-inflammatory activities of different BAPs will be discussed and summarized in Table 1.

### 4.1. Antimicrobial Activity

Antimicrobial peptides (AMPs) are a kind of BAP present in almost all life forms as part of the innate immune system, being the first defense line against a wide range of pathogens [66]. Most AMPs are characterized by a dominance of β-sheet and α-helix structures, a net positive charge ranging from +2 to +9, and a high proportion of hydrophobic amino acid residues [67]. The positive charge in AMPs is a consequence of the highly represented lysine and arginine residues, which interact with the negatively charged outer membranes of Gram-negative bacteria, leading to membrane perturbation and bacterial cell death [68,69].

In addition to the endogenous ones, AMPs are also derived from fermented foods. A significant example of food-derived AMP production is provided by the bacterium *Lactiplantibacillus plantarum*, which improves the nutritional quality, organoleptic characteristics, and antioxidant and antimicrobial activities of food, extending its shelf life and reducing the presence of undesirable compounds [70,71]. For instance, camel milk fermented with *L. plantarum* has demonstrated various health benefits, including antimicrobial, possibly due to the BAPs produced during fermentation [72]. A recent study identified a new low-molecular-weight AMP produced by *L. plantarum* FB-2, KMY15, which demonstrated a strong ability to inhibit the growth of *S. aureus* ATCC6538 and *E. coli* DH5. Furthermore, KMY15 maintained its efficacy against *S. aureus* ATCC6538, even in the presence of interfering substances such as proteins and lipids in contaminated milk [73]. In another study, antifungal peptides were released after the fermentation of kenaf seed proteins with *L. pentosus* RK3, identifying eight cationic peptides with molecular weights between 840 and 1876 Da. These peptides showed significant fungicidal activity against *Fusarium* sp. and *Aspergillus niger*, with a minimum inhibitory concentration (MIC) of 43 μg/mL and minimum fungicidal concentration (MFC) of 86 μg/mL, thus prolonging the latency phase of the fungi and increasing their membrane permeability [74]. All this evidence demonstrates the potential of AMPs as an alternative to traditional antibiotics due to their versatility, wide spectrum of activity, and the possibility of being modified to optimize their efficacy and stability under different conditions [75].

### 4.2. Antihypertensive Activity

ACE inhibitors work by blocking the enzyme ACE that converts angiotensin I into angiotensin II, a peptide that causes blood vessels to tighten. When angiotensin II is overproduced, it can lead to high blood pressure, which is a common problem in people with hypertension. Hypertension, characterized by persistently elevated blood pressure above 140/90 mmHg, is a prevalent risk factor associated with strokes and cardiovascular disease [76]. For this reason, ACE inhibitors are considered crucial for controlling blood pressure in hypertensive patients [77,78].

Recently, BAPs derived from food proteins have attracted significant interest as natural ACE inhibitors, offering a viable alternative to synthetic drugs, which often present relevant side effects [79]. Several ACE-inhibitory peptides (ACEIPs) have been isolated from fermented foods such as dairy products and vegetables [31,80,81,82,83]. In general, ACEIPs have similar primary structures, often featuring proline, tyrosine, cysteine, histidine, tryptophan, and methionine at the C-terminus, a factor that enhances their ACE inhibitory activity [84,85]. Moreover, the ACE active site contains a zinc coordination site and a hydrophobic pocket that allows interaction with aromatic residues, enhancing the binding of inhibitory peptides that contain them [86].

BAPs with all these features show strong ACE-inhibitory potential. They are likely absorbed by specific transporters across the intestinal epithelial wall, remain intact through gastrointestinal digestion, and maintain their ACE-inhibitory activities [87,88]. For instance, oral administration of the BAPs EAPLNPKANR and IVG, isolated from Cangkuk, a traditional Indonesian fermented beef, reduced blood pressure in spontaneously hypertensive rats 8 h after administration. The greatest effect was observed with IVG, due to its smaller size and the presence of isoleucine at the N-terminal, which facilitates its entry and binding to the active sites of ACE, thereby inhibiting the formation of angiotensin II and lowering blood pressure [89].

Among ACEIPs, the VPP (Valine–Proline–Proline) and IPP (Isoleucine–Proline–Proline) peptides have been extensively studied thanks to their potent ACE-inhibitory and pressure-lowering ability. These peptides were originally derived from the fermentation activity of bacteria like *L. helveticus* and yeasts like *Saccharomyces cerevisiae* in fermented milk and have since been demonstrated to yield marked antihypertensive activity in animal studies [90]. Additionally, clinical trials have shown that daily consumption of VPP/IPP tablets (10.2 mg/day) for 4 weeks decreased blood pressure levels and arterial stiffness in patients affected by metabolic syndrome [91]. Moreover, Moayedi et al. [92] used *Bacillus subtilis* to ferment tomato seeds and generate BAPs. Among the produced BAPs, the hexapeptide DGVVYY exhibited the strongest ACE-inhibitory activity, achieving an IC_50_ value of 2 µM.

### 4.3. Antioxidant Activity

BAPs derived from fermented foods have shown promising antioxidant activity by neutralizing reactive oxygen species (ROS) such as superoxide anions and hydroxyl radicals [93]. The body’s enzymatic and non-enzymatic antioxidant systems effectively neutralize harmful free radicals. However, environmental stress, lifestyle, and disease can disrupt this balance, leading to oxidative stress. This imbalance is associated with cell apoptosis and various diseases, including diabetes, atherosclerosis, and cancer [94].

It has been shown that BAPs can scavenge free radicals, reduce lipid peroxidation, and chelate metal ions, which helps prevent oxidative damage [30,95]. Their antioxidant properties are closely linked to their aminoacidic composition. For instance, peptides rich in hydrophobic amino acids have been observed to interact more effectively with cell membranes, thereby enhancing their antioxidant effects [96]. An example is represented by the peptide VLPVPQK from cheddar cheese fermented with *L. helveticus*, which contains proline residues at the N-terminus, exhibiting an antioxidant capacity of 5.71 ± 0.59 mmol of Trolox equivalents/mg in a Trolox equivalent antioxidant capacity (TEAC) assay [97]. Furthermore, pre-treatment of rat fibroblast cells with this peptide, followed by induction of oxidative stress, suppressed ROS production and increased antioxidant enzymes [98]. Another example is the peptide SNLCRPCG, derived from chicken feathers fermented with *Bacillus subtilis*, which exhibited strong antioxidant activity due to its hydrophobic amino acids and cysteine’s thiol (-SH) groups [99].

Furthermore, BAPs were found to modulate the antioxidant response by interacting with cellular signaling pathways. For instance, novel antioxidant peptides like LY-4, LP-5, and VL-9, identified from fermented broad bean paste, have been shown to activate the Kelch-like ECH-associated protein 1 (Keap1)-nuclear factor erythroid 2-related factor 2 (Nrf2) pathway, protecting liver cells (HepG2) from oxidative stress induced by 2,2′-Azobis(2-amidinopropane) dihydrochloride (AAPH), a radical generator [100]. Similarly, casein hydrolysate produced by *Lactobacillus reuteri* has been identified for its strong antioxidant potential, with the peptide VKEAMAPK showing significant effects by noncompetitively inhibiting Keap1, thereby activating the Nrf2 pathway, an essential protection mechanism against oxidative damage [101].

In another study, tomato seeds fermented with *Bacillus subtilis* produced antioxidant peptides such as GQVPP, which achieved a 97% 2,2-diphenyl-1-picrylhydrazyl (DPPH) radical scavenging activity at 0.4 mM, demonstrating its potential in reducing oxidative stress [92]. A peptide fraction with antioxidant activity was isolated and purified from whey protein metabolites fermented by *Lactobacillus rhamnosus B2-1*. The final purified peptide, B11, showed significant antioxidant activity, with a 2,2-azino-*bis*-3-ethylbenzothiazoline-6-sulphonic acid (ABTS) radical scavenging rate of 84.36%, a hydroxyl radical scavenging rate of 62.43%, and an oxygen radical absorbance capacity (ORAC) activity of 1726.44 μM Trolox equivalent/g. With an amino acid composition of 51.42%, dominated by glutamic and aspartic acids, B11 appears promising for food-based applications [102].

These findings highlight the diverse antioxidant mechanisms and health benefits of BAPs derived from fermented foods, making them promising natural alternatives to synthetic antioxidants.

### 4.4. Anti-Inflammatory Activity

The production of various immunomodulatory peptides with anti-inflammatory properties is facilitated by fermentation. They play a significant role in regulating immune response by modulating key interleukins such as IL-4 and IL-10 (anti-inflammatory), IL-1β and IL-2 (proinflammatory), IL-6 that can act as either, and cytokines such as tumor necrosis factor α (TNF-α) [103]. For instance, the hydrolysis of casein by the PrtB protease from *Lactobacillus. delbrueckii* subsp. *bulgaricus* 92,059 produced BAPs, which demonstrated immunomodulatory and anti-inflammatory effects in an in vitro assay of TNF-α-induced nuclear factor-κB (NF-κB) activation at concentrations of 5 mg/mL and 2.5 mg/mL, respectively [104]. Peptides from Xuanwei dry-cured ham have been found to reduce IL-6 and TNF-α levels in mice with dextran sodium sulfate-induced colitis, alleviating inflammatory bowel disease symptoms [105]. Moreover, soybean peptides have demonstrated beneficial effects in reducing LPS-induced intestinal inflammation in IEC-6 intestinal epithelial cells by decreasing NO production and downregulating the expression of inflammatory mediators, such as IL-1β, IL-6, and TNF-α [106].

Peptides with anti-inflammatory properties often feature positively charged and hydrophobic amino acids, especially at the C- and N-termini [107,108]. This structural composition facilitates their interaction with cell membranes and bacterial lipopolysaccharide (LPS), which is released from the wall of Gram-negative bacteria during infection and triggers inflammatory cytokines. However, the BAP’s N-terminal region can block the release of LPS, thus stopping the inflammatory response. In a study where *L. plantarum* A3 and *L. rhamnosus* ATCC7469 were used to ferment broccoli, 17 novel anti-inflammatory peptides were identified, including GDRW, KASFAFAGL, FGDFNPGGRL, and ADLAHLPF, rich in hydrophobic amino acids, and HFKQPW, RFR, and KWR, with positively charged amino acid residues [109].

**Table 1:** Summary of BAP release under specific fermentation conditions and their bioactivity.

Fermented Food	Microorganisms	Fermentation Conditions	Peptide Sequence	Bioactivity	References
Milk	*L. plantarum* FB-2	37 °C for 20 h	KMYKKGRLWLVAGLS	Antimicrobial *S*. *aureus* and *L*. *monocytogenes* MIC = 256 μg/mL *E. coli* MIC = 128 μg/mL	[73]
Milk	*L. helveticus CP790* and *Saccharomyces cerevisiae*	37 °C for 24 h	VPP IPP	ACE-I IC50 = 9 μM IC50 = 5 μM	[110]
Milk	*Lacticaseibacillus rhamnosus NCDC24*	Not specified	AGWNIPM, ALPMHIR, VLPVPQKA YLGYLEQLLR	Antioxidant ABTS+ radical scavenging activity from 73.45 ± 0.57 (100 µg/mL) to 1.44 ± 0.22 (10 µg/mL)	[111]
Miso paste	*Aspergillus oryzae*	30 °C for 40 h	VPP, IPP	Antihypertensive	[112]
Milk	*Bifidobacterium bifidum* MF20/5	37 °C for 48 h	VLPVPQK LVYPFP	Antioxidant ACE-I IC_50_ = 132 μM	[113]
Sator bean *(Parkia speciosa)*	*L. fermentum ATCC9338*	37 °C for 8 days	EAKPSFYLK PVNNNAWAYATNFVPGK	Antioxidant DPPH activity = 78.48 ± 3.16% Antibacterial activity *S. typhi* (73.41 ± 0.08%) and *S. aureus* (64.70 ± 1.10%)	[114]
Skimmed milk	*Enterococcus faecalis* CECT 5727	30 °C for 48 h	LHLPLP	Antihypertensive IC_50_ = 59.6 μg/mL	[115]
Dry fermented sausage	*L. pentosus* and *Staphylococcus carnosus*	Two stages: 20 °C for 22 h; 9 °C for 43 days	YQEPVLGPVR, YQEPVLGPVRGPFPI, YQEPLV	ACE-I IC_50_ = 300 µM	[48]
Avena *(Avena sativa* L.)	*L. plantarum B1-6* and *Rhizopus oryzae*	30 °C for 72 h	Not specified	ACE-I IC_50_ = 0.42 mg protein/mL	[55]
Lupin, quinoa, and wheat	*L. reuteri* K777 and *L. plantarum* K779	35 °C for 72 h	Not specified	ACE-I from 25.3% to 58.9% Antioxidant DPPH radical scavenging activities from 25.0% to 65.0% Antiproliferative	[56]
Wheat, soybean, Barley, and amaranth	*L. curvatus SAL33* and *L. brevis AM7*	30 °C for 16 h	Lunasin (SKWQHQQDSCRKQLQGVNLTPCEKHIMEKIQGRGDDDDDDDDD)	Cancer preventive	[57]
Budu	Not specified	Over 120 days	VAAGRTDAGVH LDDPVFIH	Antioxidant DPPH radical scavenging activity IC_50_ = 1.451 ± 0.873 (mg/mL) IC_50_ = 0.844 ± 0.203 (mg/mL)	[63]
Zebra blenny (*Salaria basilisca*) muscle protein	*Bacillus mojavensis* A21	From 4 to 48 h at 37 °C	GLPPYPYAG, LVDGLDVGIL, ETPGGTPLAPEPD, LSYEEAITTY, HHPDDFNPSVH	Antibacterial *E. coli* MIC = 0.62 ± 0.01 mg/mL *K. pneumoniae* MIC = 1.23 ± 0.02 mg/mL ACE-I Antioxidant	[62]
Pekasan (Loma fish)	*L. plantarum* IFRPD P15	2 weeks at RT	AIPPHPYP IAEVFLITDPK	Antioxidant activity DPPH radical scavenging activity IC_50_ (mg/mL) = 1.38 ± 0.25 IC_50_ (mg/mL) = 0.897 ± 0.84	[64]
Manila clam *(Ruditapes philippinarum)*	*Bacillus natto*	37 °C for 36 h	VISDEDGVTH	ACE-I IC_50_ = 8.16 μM	[18]
Thai shrimp pastes	Not specified	Not specified	SV, IF, WP	ACE-I IC_50_ = 60.68 ± 1.06 μM Antioxidant ABTS^+^ EC_50_ = 17.52 ± 0.46 μM	[65]
Kenaf seed	*L. casei*	37 °C for 72 h	AKVGLKPGGFFVLK, GSTIK, LLLSK, TAHDDYK	Antibacterial activity from 42.07% to 77.38%	[116]
Tomato waste proteins	*Bacillus subtilis*	37 °C for 24 h	DGVVYY GQVPP	ACE-I IC_50_ = 2 µM Antioxidant 97% DPPH scavenging activity at 0.4 mM	[92]
Cheddar cheese	*L. helveticus* and *Streptococcus thermophilus*	Not specified	EMPFPK, AVPYPQR, VLPVPQK, AMKPWIQPK	Antioxidant TEAC = 5.7 ± 0.6 mmol TE/mg	[97]
Feather hydrolysate	*Bacillus subtilis S1-4*	37 °C for 72 h	SNLCRPCG	Antioxidant DPPH IC_50_ = 0.39 mg/mL	[99]
Whey protein	*L. rhamnosus B2-1*	37 °C for 48 h	B11	Antioxidant ABTS^+^ radical scavenging activities = 84.36%	[102]
Casein	*L. reuteri*	Not specified	VKEAMAPK	Antioxidant Decreased ROS activity by 45%	[101]
Broccoli	*L. plantarum A3* and *L. rhamnosus ATCC7469*	37 °C for 24 h	SIWYGPDRP	Anti-inflammatory Inhibits NO release from inflammatory cells at 25 µM, with an inhibition rate of 52.32 ± 1.48	[109]

RT: room temperature. ACE-I: angiotensin-converting enzyme-inhibitory activity. TEAC: Trolox equivalent antioxidant capacity. ROS: reactive oxygen species. Each letter in the peptide sequence corresponds to an amino acid: A = alanine, C = cysteine, D = aspartic acid, E = glutamic acid, F = phenylalanine, G = glycine, H = histidine, I = isoleucine, K = lysine, L = leucine, M = methionine, N = asparagine, P = proline, Q = glutamine, R = arginine, S = serine, T = threonine, V = valine, W = tryptophan, Y = tyrosine.

## 5. Challenges and Limitations in BAPs Production

The development of BAPs is a promising yet challenging area in biotechnology, with potential applications across the food, nutraceutical, and pharmaceutical industries. However, several significant obstacles impede their efficient development and commercialization. One of the foremost challenges in developing BAPs is the scalability of production processes. In microbial fermentation, issues like longer reaction times and low yield of peptide formation pose additional barriers. The complex proteolytic systems in different microorganisms result in variations in peptide structures, making it difficult to standardize production processes [24]. Even among the same species of bacteria, such as *Lactobacillus* or *Bacillus*, differences in proteolytic capabilities can result in different peptide profiles when exposed to the same substrates [117]. While enzymatic hydrolysis offers more controlled conditions, microbial fermentation involves a longer reaction time and greater unpredictability in the peptides generated. Factors such as the type of microorganism, the protein substrate used, fermentation time, and environmental conditions (e.g., pH, temperature, and oxygen levels) all impact the extent and specificity of hydrolysis [118].

Additionally, the purification of BAPs from fermented mixtures is technically demanding and costly. Microbial fermentation typically yields a complex mixture of peptides, alongside other microbial by-products such as exopolysaccharides, bacteriocins, and dead microbial cells. These additional compounds can interfere with the isolation of pure BAPs and complicate the identification of the specific bioactive properties of the peptides [119]. Purification processes are essential to isolate peptides with specific biological activities, yet they are time-consuming and costly. Ultrafiltration and chromatographic techniques, such as HPLC, are commonly used but are often not suitable for large-scale production due to issues like membrane fouling and poor reproducibility [120]. Once produced and purified, BAPs face challenges related to their stability and bioavailability, covered in depth in Section 6.

Furthermore, regulatory approval and clinical validation hinder the production of BAPs through microbial fermentation. Despite the promising bioactivities demonstrated in in vitro and animal studies, there is a lack of well-designed clinical trials to confirm the efficacy of food-derived peptides in humans [121]. Regulatory agencies, such as the European Food Safety Authority (EFSA), require rigorous evidence of the safety and efficacy of functional food ingredients before they can be marketed. Without sufficient clinical evidence, it is difficult for companies to commercialize BAPs or make health claims about their benefits, which hinders their marketability [5]. Therefore, addressing these issues is critical for the future success of BAPs in the health and wellness industries.

## 6. Approaches for Enhancing BAP Production, Stability, and Bioavailability

**Figure 2:**
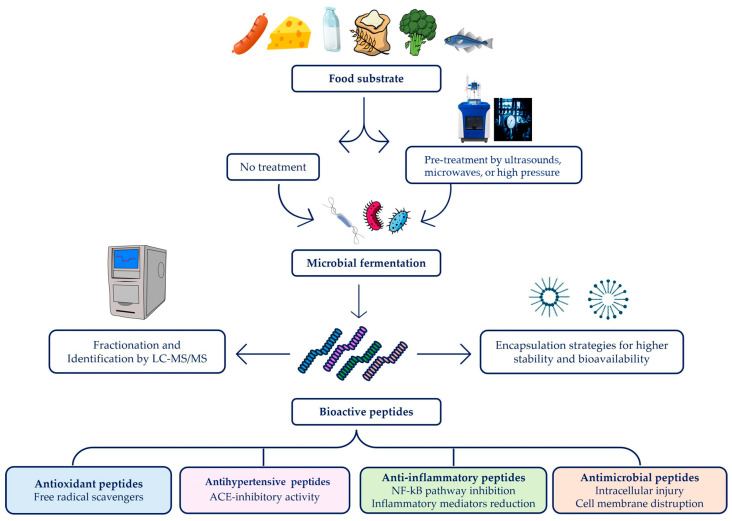
Schematic representation of BAP production and their associated bioactivities.

Since the fermentation process often results in low yields of BAPs, besides modulating physicochemical parameters such as temperature, pH, and time, emerging green technologies and encapsulation strategies have been employed to enhance BAP content, stability, and bioavailability. Figure 2 summarizes BAP production techniques, stability/bioavailability approaches, and the major mechanisms responsible for BAP biological activity.

### 6.1. Strategies to Optimize the Production of BAPs in Fermented Foods

Microbial fermentation has been extensively used to improve the nutritional quality and health-promoting properties of various foodstuffs, for instance, by increasing the availability of BAPs. However, despite being economical and easy to perform, fermentation displays low yields of BAP production, a factor that limits its industrial escalation [9,122]. Thus, several strategies have been attempted to promote protein hydrolysis and increase the recovery of BAPs.

One such way consists of exposing the food matrix to ultrasounds, microwaves, or high pressure before the fermentation process [9,122]. In a study performed by Munir and colleagues [123], Cheddar cheese was produced with milk pre-treated by either ultrasonication, high-pressure processing, or microwave, and its antioxidant and ACE-inhibitory activities were followed during ripening.

It was revealed that the pre-processing steps increased the rate of proteolysis during cheese making and ripening, as well as the antioxidant and ACE-inhibitory activities of the resulting cheese, with the best results obtained with ultrasonication. Additionally, moderate ultrasound exposure during fermentation has been proven to affect the activity of microorganisms, as testified by the work of Xie et al. [124]. Indeed, they showed that ultrasound-assisted fermentation of okra improved its peptide and soluble protein contents, enhancing its antioxidant properties.

Finally, the food matrix can also be processed after fermentation to increase the yield of BAPs. As an example, milk fermented with *Lactobacillus delbrueckii* QS306 and subjected to ultrahigh-pressure treatment exerted a higher peptide concentration and variety, and an increased ACE-inhibitory activity compared to the non-processed sample [125].

Another strategy to enhance BAP yield is to optimize the fermentation parameters, including incubation time, temperature, pH, and inoculation rate to find the optimal conditions. Khakhariya et al. [126] explored this approach by studying the production of health-promoting BAPs in fermented buffalo and camel milk by *Limosilactobacillus fermentum* (KGL4) and *Saccharomyces cerevisiae* (WBS2A). They monitored the variation in ACE-inhibitory, antidiabetic, and proteolytic activity at 37 °C and at multiple time intervals (12, 24, 36, and 48 h) and inoculation rates (1.5%, 2.0%, and 2.5%), finding the optimal fermentation conditions to be a 2.5% inoculation rate and 48 h. One investigation on BAPs in flaxseed milk fermented with *L. plantarum* (NCDC 374) revealed that the optimal conditions to guarantee the best bioactivity (antioxidant, ACE-inhibition, and proteolysis) were a 4.20% inoculum size and 126 h of fermentation time [127]. Additionally, it was shown that pH is critical to optimizing BAP retrieval and bioactivity in fermented foods. For instance, the functional properties and BAP production in lentils fermented using *L. plantarum* combined with a commercial protease (Savinase^®^ 16 L) benefited from a mild alkaline environment. A multivariate analysis highlighted a pH of 8.5 and 11.6 h of incubation time as the optimum [128]. Taken together, these studies highlight the possibility of improving the fermentation performance of foods, maximizing BAP release.

### 6.2. Strategies to Improve the Stability and Bioavailability of BAPs

Improving the stability and bioavailability of BAPs is essential for maximizing their health benefits in functional foods, nutraceuticals, and pharmaceuticals. Despite their promising biological activities, BAPs face several challenges that limit their application, particularly their susceptibility to enzymatic degradation, low bioavailability, and poor stability under various physiological and processing conditions [129]. Several advanced strategies have been developed to address these limitations, including encapsulation technologies, carrier matrices, and physicochemical modifications [10,130].

One major challenge in enhancing BAPs’ stability and bioavailability is their susceptibility to gastrointestinal degradation. Peptidases in the stomach and intestinal lumen degrade BAPs, reducing their ability to reach the bloodstream in their active form [131]. Encapsulation is a commonly employed technique for maintaining bioactive compounds’ functional and physicochemical properties. It involves surrounding solid or liquid particles with a coating or embedding them in a matrix [132].

Several materials can be used as carriers for peptide encapsulation, including proteins, polysaccharides, and lipids. Each type of carrier offers distinct advantages and faces specific challenges. Proteins, for instance, possess valuable functional properties, such as emulsification, water retention, and the ability to form gels, making them suitable candidates for encapsulation [133,134,135].

Polysaccharides are another promising class of carrier materials for encapsulation due to their structural stability, abundance, and low cost [136]. They have reactive functional groups that can form interactions with BAPs, helping to stabilize and protect them during processing and storage [132]. Polysaccharides such as starch, dextrin, gum arabic, pectin, and chitosan have been widely used in peptide encapsulation [132,137]. Their use is often combined with proteins to achieve better encapsulation efficiency and bioactive compound stabilization [131,138].

Lipid-based carriers, such as liposomes and nanoliposomes, have also gained significant attention as good systems for BAPs’ encapsulation [132]. Liposomes consist of phospholipid bilayers, which can be useful to encapsulate both hydrophilic and lipophilic substances [131,139]. This versatility makes liposomes an attractive alternative for delivering a wide range of bioactive compounds. Nanoliposomes, which are smaller in size, provide even better protection and improved bioavailability by facilitating the controlled release of encapsulated peptides [140,141]. For instance, BAPs derived from shrimp waste have been stabilized through chitosan-coated nanoliposome formation [142].

Among the methods for peptide encapsulation, spray drying is a popular method for encapsulating bioactive compounds, including peptides, because it is economical, flexible, and capable of producing stable powders that can be easily incorporated into food products. During the spray drying process, peptides are mixed with carrier materials that form a protective film around the peptide [132]. This approach prevents peptide degradation during processing and storage but also effectively masks undesirable flavors, such as bitterness, as demonstrated by Sarabandi et al. [134] through spray drying encapsulation to stabilize peptides from oleaster seeds.

Another method is nanoencapsulation, which represents a promising approach for improving peptide stability and bioavailability. Nanoparticles can be engineered to protect peptides from degradation, enhance their absorption in the intestine, and ensure their targeted delivery to specific tissues [143]. Nanoparticles made from materials such as dextran, chitosan, or amphiphilic molecules can improve the bioaccessibility and controlled release of peptides. These nanoscale systems offer several advantages over traditional encapsulation methods, including increased surface area, enhanced cellular uptake, and the ability to penetrate physiological barriers [144].

Physicochemical modifications of peptides such as the Maillard reaction (MR) also hold the potential for improving their stability and bioavailability. The MR involves the interaction between amino acids and reducing sugars, which leads to the formation of peptide–sugar conjugates [145]. These conjugates have been shown to enhance the stability of peptides during processing and storage and improve their taste and overall sensory properties. Additionally, MR-conjugated peptides may exhibit enhanced biological activities, such as antioxidant or antimicrobial effects, further increasing their potential as functional ingredients in foods and nutraceuticals [130].

Therefore, improving the stability and bioavailability of BAPs is crucial for their successful incorporation into functional foods, nutraceuticals, and pharmaceuticals. Encapsulation and physicochemical modifications are among the most promising strategies for achieving these goals.

## 7. Conclusions and Future Perspectives

By reviewing a substantial slice of the literature, we have confirmed microbial fermentation as a valuable natural technology extensively used to enrich several animal- and plant-derived foods with BAPs. This approach offers structurally variable peptides due to the diversity of microbial proteases but also supports a wide range of beneficial biological activities, namely antimicrobial, anti-inflammatory, antioxidant, and antihypertensive. Adopting innovative technologies such as ultrasound, microwave, and high pressure, combined with optimizing fermentation parameters, represents a promising avenue for scaling up this process. Additionally, finding appropriate delivery strategies is crucial for guaranteeing the stability and efficacy of BAPs. As research and fermentation technologies continue to advance, new perspectives are emerging for developing functional products with high added value. This will also be achieved by establishing interdisciplinary collaborations, involving food technologists, microbiologists, biochemists, and medical professionals, ensuring the scalability of BAPs’ production processes.

## Data Availability

The original contributions presented in the study are included in the article, further inquiries can be directed to the corresponding author.
